# Approaches towards pragmatic language assessment in Indian pre-schoolers: A survey among speech-language pathologists

**DOI:** 10.12688/f1000research.154514.1

**Published:** 2024-08-01

**Authors:** Saniya Rasheeka, Sudhin Karuppali, Jayashree Bhat, Megha Mohan, Aiswarya Varghese

**Affiliations:** 1 Audiology and Speech Language Pathology, Kasturba Medical College Mangalore, Manipal Academy of Higher Education, Mangalore, Karnataka, 576104, India; 2 Department of Audiology and Speech Language Pathology, Nitte Institute of Speech and Hearing, Deralakatte, NITTE (deemed to be University), Mangalore, Karnataka, 575018, India

**Keywords:** assessment, barriers, facilitators, pragmatic language, preschoolers, self-appraisal, speech-language pathologists, survey

## Abstract

**Background:**

Pragmatic language assessment in children is performed in line with standard protocols, guidelines, and best practices. The absence of these aspects in the Indian context has resulted in the quest to explore the approaches used by speech-language pathologists (SLPs) to assess pragmatic language impairments. This survey explored the current practices of SLPs towards the assessment of pragmatic language among preschool children in India. It also aimed to identify the barriers, facilitators and identify the level of knowledge, skill and overall practice of SLPs towards their practices using self-appraisal.

**Methods:**

A total of 100 SLPs(94 females and 6 males) working with preschool aged children (three-to-six-year-olds) from across different Indian states participated in the survey. Participants were enquired about the aspects of pragmatic language assessed, methods used for assessment, awareness and use of Indian tools, the settings, members, and language used for the assessment. Additionally, they were asked to mention the specific tools used, informal methods used, barriers and facilitators, and self-appraise their knowledge, skill and overall practice.

**Results:**

Majority of participants assessed multiple aspects of pragmatic language. All used a combination of different assessment methods, with the participants commonly using informal compared to formal approaches. Preschoolers were assessed at multiple settings, along with different communication partners. Lack of awareness on assessment tools developed in India was the major barrier, while the use of informal tasks or activities were the major facilitators influencing pragmatic language assessment to a greater extent. The knowledge and skills for the assessment of pragmatic language obtained poorer scores compared to practices.

**Conclusions:**

The assessment practices of the SLPs were largely influenced by the unavailability of developed or adapted tools for Indian preschoolers, leading to the need to develop indigenous assessment tools. Certain considerations for further assessment practices have been identified and discussed.

## Introduction

Pragmatic language, often referred to as ‘pragmatics’ is the ability to apply language resources to facilitate effective social interaction, considering the needs of the conversational partner and demands of the physical context ( Stephens & Matthews, 2014). It encompasses the unspoken rules and ways of making communication appropriate and effective within specific contexts. This primarily includes the understanding and using of language for different purposes, such as to comment, direct, reject, protest, greet, inform, demand, state, promise, request, etc. Secondly it comprises of changing language according to the listener needs (considering partner’s age and relationship) such as (a) talking appropriately to unfamiliar individuals, (b) shifting registers, using stylistic variations according to the age of the communication partner, (c) using polite forms such as ‘please’ during requests especially when the communication partner is older or less familiar and in a dominant role, (d) being extra polite when demanded, and(e) using indirect requests. It also includes changing language according to situations, using a different tone or altering the choice of words in the classroom when compared to the playground. Lastly, pragmatics includes following the rules of the conversation such as, initiating a conversation with a question/statement, introducing new topics, maintaining the topic, taking turns, seeking for clarification, repairing a communication breakdown, clarifying, and organizing the structure for different discourse genres like narration and expository ( Cummings, 2014; Matthews et al., 2018).

Pragmatic competence requires the sophisticated coordination between linguistic ( Vogindroukas et al., 2021), social ( Šimleša & Cepanec, 2015), and cognitive skills ( Papafragou, 2018; Toe et al., 2020). Owing to the parallel development of these skills, pragmatic language is acquired early in childhood, playing a pivotal role through preschool years (3 to 6 years) ( Fahey et al., 2019). The emergence and refinement of social communication skills during this period ( Alduais et al., 2022), lays a foundation for building relationships as children begin to form bondswith peers, neighbours, teachers, and others apart from the immediate family ( Dillon et al., 2021). With preschool entry, children acquire basics of classroom behaviour such as greeting, listening, asking questions, following group instructions, thereby preparing to navigate themselves through academic-social environments. They begin to assert independence, express likes and dislikes through self-exploration and expression, thus developing a sense of identity and autonomy. These children identify, express, and manage emotions thereby effectively communicating feelings, asking for help, or offering comfort to others, thereby acquiring knowledge about the world through social interactions. Everyday conversations provide ample opportunities for expanding vocabulary, understanding complex concepts, and building existing knowledge ( Clark, 2018). Pragmatic skills such as negotiating in order to settle disagreements among peers, problem-solving skills to resolve social challenges become a part of a preschooler’s life. In essence, the development of pragmatic language helps preschoolers navigate the social world, build relationships, and lay the groundwork for future academic and social success ( Toe et al., 2020).

A delay or impairment in the pragmatic language during childhood results in long-term negative impact on the child’s personal, academic, social and various other aspects of life ( Dillon et al., 2021; Elleseff, 2015; Loukusa et al., 2018). Reports of a growing trend in neuro developmental disorders have resulted in an increase of language impairments encountered by speech-language pathologists (SLPs) all over the world ( American Speech-Language-Hearing Association, 2022). A high prevalence of language impairments among Indian children have been reported ( Konadath et al., 2013, 2017; Shanbal et al., 2015; Sinha et al., 2017), that isinclusive of certain disorders explicitly diagnosed based on pragmatic language characteristics ( Swineford et al., 2014). Pragmatics forms the central feature in identifying Autism Spectrum Disorders (ASD) ( Hage et al., 2022; Wong et al., 2022). An increase in the prevalence rates (89 per 10,000) of ASD in India ( Poovathinal et al., 2016) has raised concerns among SLPs ( Dillon et al., 2021). Given that the majority of children are not diagnosed with ASD until or after the age of 4 or 5 years ( Centers for Disease Control and Prevention, 2019), or median age of 4.5 years ( Brett et al., 2016), SLPs are more likely to encounter preschool age children with ASD symptoms whose diagnosis are awaited ( Binns et al., 2022). Furthermore, fifth edition of Diagnostic and Statistical Manual of Mental Disorders ( American Psychiatric Association, 2013) included a definite diagnostic criteria for social (pragmatic) communication disorder as an independent addition characterized exclusively on pragmatic abilities. This has further exemplified the need to identify impairments related to pragmatic language among children by using suitable assessment measures ( Salari et al., 2022). However, the understanding of developmental norms ( Gerber et al., 2012; Izaryk et al., 2021; O’Neill, 2014) including the clinical methodologies and intervention approaches ( Ash et al., 2017; O’Neill, 2014; Swineford et al., 2014) are still in its infancy.

Globally, there exists best practices in the assessment and intervention of various neurodevelopmental disorders resulting in pragmatic deficits ( Adams et al., 2015; Elleseff, 2015; Timler, 2018). However, in India, there has been an inadequate amount of information guiding SLPs towards the same, despite the increase in the prevalence of pragmatic language impairments and the need for early identification and timely intervention. The absence of standard protocols, guidelines, and/or best pragmatic language assessment tools does result in inconsistencies among practicing SLPs, leading to the poor identification of impairments, and the lack of confidence in one’s own practice. In the face of such challenges, it becomes important to understand the approaches used by SLPs to identify and assess pragmatic language impairments in children. Therefore, the first and foremost initiative is to explore the current practices ( Binns et al., 2022) based on the nature of pragmatic assessment (‘what is being assessed in pragmatics?’, ‘how has it been assessed?’, ‘where to assess?’, ‘what tools and materials are available or can be used?’, and many more). Such questions require collective responses to find the most prevalent and benefitting practices that can be consequently imbibed during the assessment of pragmatics among preschoolers. Therefore, the primary aim of the current study was to explore the current SLP practices towards the assessment of pragmatic language among Indian preschoolers using a survey method. Most survey studies lack the SLPs’ perspectives towards the barriers and facilitators to optimal service delivery, as well as their self-assessment of knowledge, skills, and overall practice. It therefore becomes critical to understand clinicians’ perspectives to ensure the development of assessment and/or interventions enabling optimal service delivery ( Binns et al., 2022). Self-appraisal forms a fundamental skill required throughout learning and constant self-performance evaluation in an SLP’s career ( Yan, 2020). Therefore, the current study also aims to identify the barriers and facilitators towards the assessment of pragmatic language among preschoolers and identify the level of knowledge, skill and overall practice of SLPs towards their own assessment routines.

## Methods

A cross-sectional online survey design was adopted for the present study as it aimed to obtain an understanding of the assessment practices among SLPs across the country. The study was approved by the Institutional Ethics Committee of Kasturba Medical College, Mangalore, Manipal Academy of Higher Education, India as a part of a doctoral study (IEC KMC MLR 12-2020/418) on 18-03-2021. Informed consent was obtained from the participants by voluntarily placing a tick (✓) on the checkbox prior to participation in the study since the survey was conducted online using Google Forms.

### Survey questionnaire

The questionnaire was developed by the authors to survey SLPs’ current approaches towards assessment of pragmatic language among preschoolers in India. Section one of the questionnaire involved demographics, work setting, and caseload details of the participant (SLP), while section two involved forced-choice questions on pragmatic language assessment practices in preschoolers. Section three targeted the barriers and facilitators in pragmatic language assessments and its influence on the same ( Damschroder et al., 2022), using a four-point Likert rating scale [1–barrier/facilitator to no extent (alternatively meaning, not a barrier/facilitator), 2–barrier/facilitator to a little extent, 3–barrier/facilitator to a moderate extent, 4–barrier/facilitator to a great extent] ( Kajermo et al., 2010). Section four targeted the participant’s knowledge, skill, and overall practice in pragmatic language assessments using a five-point Likert rating scale of quality (1–poor, 2–below average, 3–average, 4–good, 5 – excellent). The presence of various question types such as filling in the blank statements, rating scales, forced-choice, multiple-response, and open-ended questions were deemed to capture the comprehensive practices followed by the participants ( Binns et al., 2022). Considering the limitation of using forced-choice and multiple-response question types ( Gillon et al., 2017), aprovision of reporting any other type of descriptive responses beyond the choices presented in the questionnaire was also included ( Binns et al., 2022; Izaryk et al., 2021). Lastly, the questionnaire concluded with an opportunity for the participants to provide any additional remark pertaining to their practices.

The initial version of the questionnaire was subjected to content validation by five SLPs on a five-point Likert rating scale for item relevance (from 1 being irrelevant to 5 being highly relevant) ( Polit & Beck, 2006). Additionally, feedback and comments were sought for each item in the questionnaire. The Content Validation Index (CVI) for the overall item level CVI scores ranged between 0.9-1.00 and scale-level CVI scores for each section was 1.00, both indicating excellent content validity ( Polit et al., 2007). Feedback from the content validation was integrated, and the final questionnaire ( Rasheeka et al., 2024) was ready to be piloted among six SLPs to determine the item clarity, logical sequence of questions, and the response options across the questionnaire.

### Survey participants

Potential participants received the survey information sheet with the link to the online questionnaire via one of two modes: (a) posting a research participation request on the Indian Speech-Language and Hearing Association (ISHA) website ( www.ishaindia.org.in), and (b) posting research requests on professional groups and social networking platforms such as WhatsApp and Instagram. A snowball sampling method was followed to aid wider distribution of the survey to potential participants. The online link to the survey was open for 60 days (from 1 ^st^ August to 30 ^th^ September 2023), and the responses were collected through Google Forms (a web-based platform). Multiple participation from the same participant was prevented by turning on the ‘limit to 1 response’ option on Google Forms. A total of 100 (94 females and six males) SLPs registered under the Rehabilitation Council of India (RCI) participated in the survey. The participants worked with preschool aged children (3 to 6 years) at various settings within India in the last six months. The respondents were from Karnataka (n=52), Kerala (n=21), Tamil Nadu (n=14), Maharashtra, Gujarat, Rajasthan (n=2in each), Pondicherry, Delhi, Haryana, West Bengal, Assam, Bihar, and Telangana (n=1in each). The participants took 15 minutes on average to complete the survey. Table 1 shows the demographic details of the current study participants.

**Table 1.** Demographic details of survey participants.

Demographics	Mean	SD	Range	Median	Mode
Age (in years)	27.91	5.66	22 – 50	26	24
Experience (in years)	4.6	5.37	1 – 30	2	1
Language assessments per month	23.05	40.44	2 – 300	10	10

Demographics	Characteristics	Frequency ( *n =*100)
Qualification	Bachelor’s	41
	Master’s	58
	Doctorate	1
Work setting	Private clinic or rehabilitation centre	56
	Institution/university affiliated clinic	18
	Hospital	19
	School	3
	Telerehabilitation	4
Service provision	Children	47
	Children, adults	53
Community	Rural	9
	Urban	91

### Data analysis

The responses were subjected to quantitative and qualitative analysis based on the type of survey question. Descriptive statistics was employed using Jamovi version 2.4.8. For questions with multiple item responses or with a large number of responses, the options were grouped into overarching categories ( Lal et al., 2022). For open-ended questions (such as the use of different formal assessment tools, informal tasks or activities, and materials), the responses were analysed using content analysis and grouped into main categories ( Braun & Clarke, 2006). As it was not mandatory to respond to all open-ended questions, the data was reported as a percentage of the number of responses received (indicated by denominator). Subgroup analysis and data triangulation across questions was used to elucidate further information ( Lal et al., 2022). The qualitative analysis of the obtained responses regarding the assessment practices were important in corresponding with the obtained quantitative data ( Gillon et al., 2017).

## Results

The survey results provided a comprehensive overview of SLPs’ pragmatic language assessment practices, approaches, barriers, facilitators and self-appraisal in terms of knowledge, skills, and overall practice.

### Approaches to pragmatic language assessment

i. *Caseload representation and choice of pragmatic language assessment*

Most of the SLPs’ case load included a greater percentage of preschoolers with ASD, Delayed Language Development (DLD) (collective term used for children with primary difficulties in language), and Attention Deficit Hyperactivity Disorder (ADHD). The assessment of pragmatic language among these language disorders have been reported to be at a higher rate when compared to preschoolers with diagnoses such as Global Developmental Delay (GDD), Intellectual Disability (ID), Down's syndrome (DS), Cerebral Palsy (CP), Cleft Lip and Palate (CLP), or any other. The caseload representation and proportion of each diagnosison whom pragmatic language assessment was conducted by the study participants are presented in Table 2.

**Table 2.** Study participants’ caseload representation and their pragmatic assessment routine.

Sl. No	Diagnosis	Encountering preschoolers with the diagnosis (%)	Assessment of pragmatic language	
			Performed by participants [% (n)]	Not performed by participants [% (n)]
1.	ASD	95	73.68 (70)	26.32 (25)
2.	DLD	95	69.47 (66)	30.53 (29)
3.	ADHD	85	71.76 (61)	28.24 (24)
4.	GDD	67	46.27 (31)	53.73 (36)
5.	ID	64	59.38 (38)	40.62 (26)
6.	DS	51	43.14 (22)	56.86 (29)
7.	CP	45	35.55 (16)	64.44 (29)
8.	CLP	36	36.11 (13)	63.89 (23)
9.	Others	20	45 (9)	55 (11)

Note: ASD - Autism Spectrum Disorder, DLD - Delayed Language Development, ADHD - Attention Deficit Hyperactivity Disorder, GDD - Global Developmental Delay, ID - Intellectual Disability, DS - Down's syndrome, CP - Cerebral Palsy, CLP - Cleft Lip and Palate.

Fifty-three percent of the participants reported performing a routine assessment of pragmatic language on every preschool aged child in their caseload, of which 64.15% (34/53) performed screening, 24.53% (13/53) performed screening and diagnostic assessment, and 11.32% (6/53) performed a diagnostic assessment. Forty-seven percent of the participants assessed for pragmatic language on a part of their caseload, of which 78.72% (37/47) performed only screening, 14.89% (7/47) performed screening and diagnostic assessment, and6.38% (3/47) performed a diagnostic assessment.

ii. *Assessment of different aspects of pragmatic language*

All study participants assessed multiple aspects of a preschooler’s pragmatic language. Forty-three percent of the participants assessed all the five aspects of pragmatic language (see Table 3). Twenty-two percent of the participants assessed a combination of three aspects, preschooler’s way of using language for different purposes (greeting, thanking, informing, requesting, etc.), conversation and narrative abilities. Additionally, the ‘other’ option category received responses such as identifying play behaviours, language used during play, awareness of body proximity, intention to communicate, participation in social interaction, expression of emotions and desires, interpretation of humour, theory of mind and child’s personality. While all these additional responses targeted on profiling the child’s pragmatic language abilities, one of the responses emphasized on identifying the parent’s role and their interaction style during a conversation with the preschooler.

iii. *Methods to assess pragmatic language*

**Table 3.** Different aspects of pragmatic language assessed by the study participants.

Sl. No	Aspects of pragmatic language assessed	Participants (%)
1.	Way of using language for different purposes (greeting, thanking, informing, requesting, etc.)	94
2.	Conversation abilities	86
3.	Narrative abilities	72
4.	Way of adapting language according to listener (considering partner’s age and relationship)	52
5.	Way of adapting language according to situation (using a different tone or altering/varying the choice of words in the classroom versus/when compared to the playground)	43
6.	Other	7

**Figure 1.**
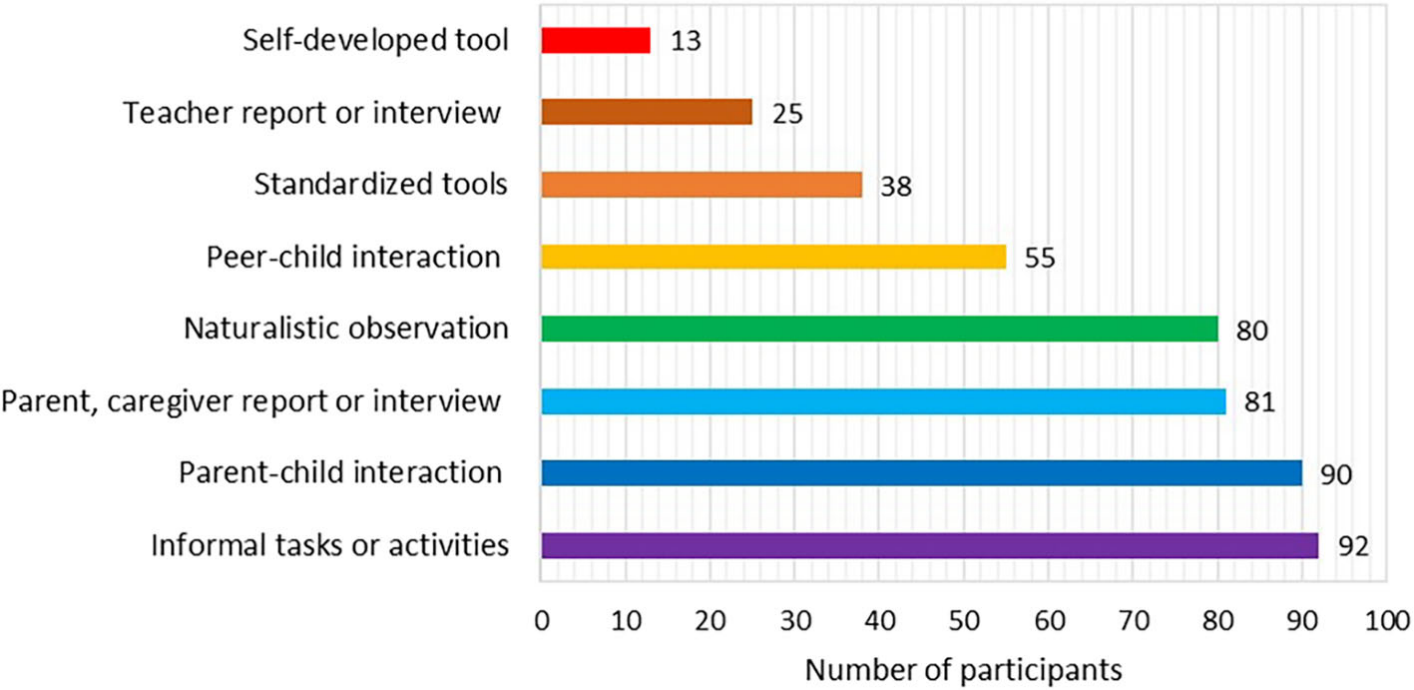
Methods to assess pragmatic language as reported by the study participants.

Informal tasks or activities were used by majority (92%) of the participants, followed by parent-child interaction (90%) and other methods (see Figure 1). Most participants (98%) used a combination of different assessment methods except for two of them who used only informal tasks or activities during an interaction with preschoolers. The most frequent number of methods combined by the participants was 5 ( *M* = 4.74, *SD* = 1.43, range: 1-8) and the commonly reported (15%) combination of assessment methods included, peer-child interaction, observation of child in natural settings, parent or caregiver report or interview, parent-child interaction, and informal tasks or activities during the interaction with preschoolers. The next most reported (12%) combination included the same methods except for peer-child interaction.

**Figure 2.**
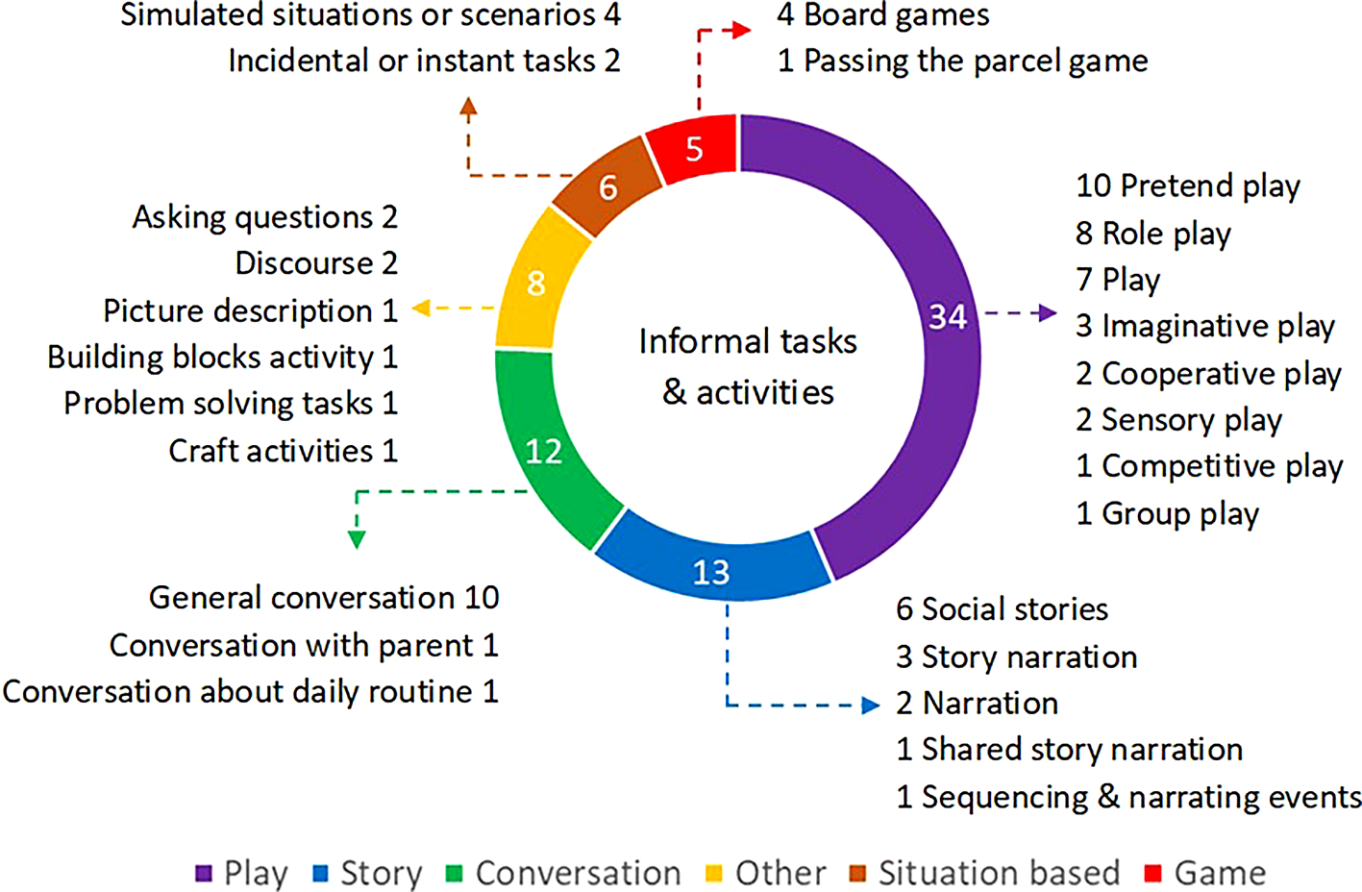
Categories and subcategories of informal tasks and activities used for the assessment of pragmatic language.

Participants were further enquired about the different tasks or activities used as a part of informal approach, and the different tools used as a part of formal or standardized approach to assessment. As the probe questions were optional, the results were based on the number of responding participants.

a. *Use of informal tasks and activities:* The various informal tasks and activities reportedby34 participants were broadly classified into six categories. Different kinds of play were mentioned repeatedly by most SLPs ( *n* = 34). Figure 2 provides a visual representation of the frequency of categories and subcategories of the tasks and activities used for the assessment of pragmatic language among preschoolers.

**Figure 3.**
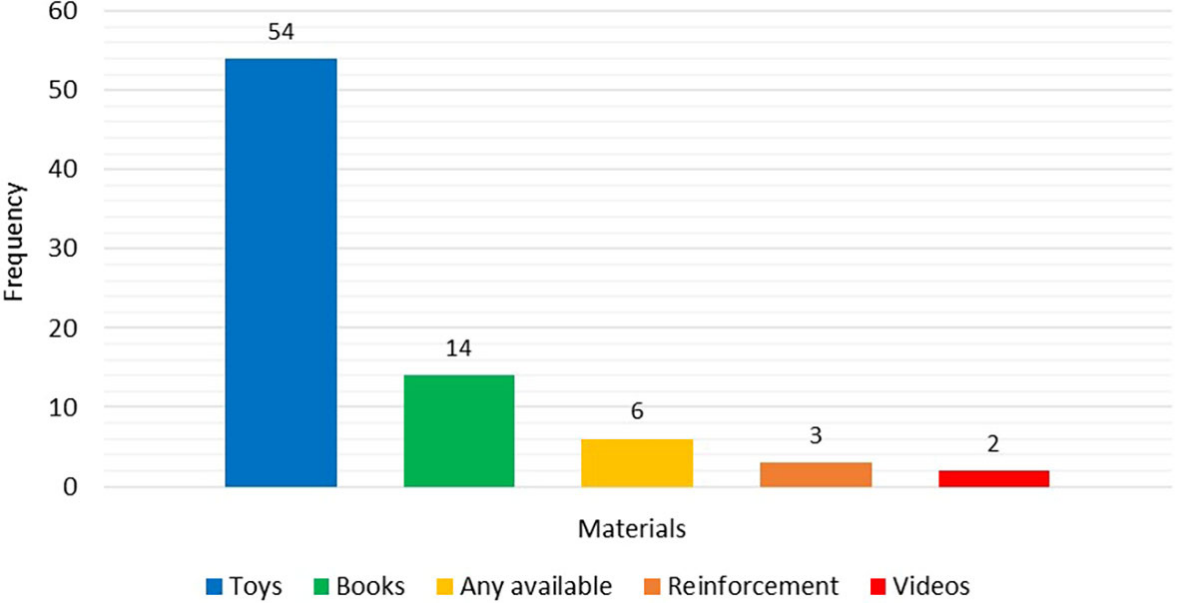
Frequency of different materials mentioned as being used during the assessment of pragmatic language.

Furthermore, participants were enquired about the different materials (or objects) used during the assessment. The various materials mentioned by 38 SLPs were broadly classified into five categories. Toys were reported more frequently (54), followed by books (14) and other materials. Figure 3 provides a visual representation of the different materials reported of being used by the survey participants for the assessment of pragmatic language among preschoolers.

b. *Use of assessment tools:* Twenty-seven participants explicitly mentioned the standardized tools used for the assessment of pragmatic language among preschoolers (see Table 4). Out of which, 23 distinct assessment tools were identified which included those specifically designed for pragmatic language (10), comprehensive language (9), and other unidentifiable tools (4). It was reported that participants did not necessarily administer the entire tool for the assessment of pragmatic language (mentioned in the table), rather used relevant sections from certain available tools. The use of pragmatics profile section from Clinical Evaluation of Language Fundamentals (CELF) - 4 ^th^ and 5 ^th^ edition was reported by most (7) of the SLPs, followed by Speech and Language Development Chart (SLDC) (4), Pragmatics Profile of Everyday Communication Skills in Children (PPECS) (4) and other tools. In addition to the reported tools, one of the participants stated utilizing sections of DSM-5 for the assessment of pragmatic language.
c. *Awareness on Indian tools for pragmatic language assessment for preschoolers:* Among the total100 participants, only a few (16%) reported of being aware of and having access to Indian developed assessment tools, of which six of them listed Communication DEALL Developmental Checklist (CDDC), Checklist for Assessment of Pragmatics in Preschoolers (CAPP), Preschool Language Scales – 5 ^th^ edition (PLS) ( Zimmerman et al., 2011), Test of Pragmatic Language – 2 ^nd^ edition (TOPL) and two other unidentifiable assessment tools (Test of Early Milestones and PLI). Of the mentioned tools, only two (CDDC, CAPP) were developed for Indian population. Twenty-three participants reported of being aware, but having no access to Indian developed assessment tools, of which four listed CDDC and two used tools (Pragmatic assessment test and Pragmatic assessment tool) that were unidentifiable for the purpose of the study. Nevertheless, there was a significant number of participants (61%)who were unaware of any Indian developed tools for the assessment on pragmatic language for preschoolers.

iv. *Setting for the pragmatic language assessment for preschoolers:* Majority of the SLPs reported of performing an assessment of pragmatic language at multiple settings, with 42% of them assessing at the testing/consultation room, child’s home, and preschool environment;47% assessing at the testing/consultation room, and preschool environment; and56% assessing at testing/consultation room, and the child’s home environment. On the other hand, participants assessed in single settings [at home (2%), and their testing/consultation room (34%)]. While few participants reported of using videos of the child in different environment, others reported otherwise. For e.g., one of the participants mentioned “I don’t actually go personally to child’s home or school and assess; rather, I obtain information from different environments using parent interview or report”. One of the participants reported of conducting assessment in children’s play area.
v. *Members involved in the pragmatic language assessment for preschoolers:* During the assessment of pragmatic language among preschoolers, involvement was reported from parent/s and/or caregiver/s (98%), siblings (70%), co-clinicians (57%), peers (49%), and teachers (38%). Twenty-two percent of the SLPs reported involving all the five members during the assessment in their practice, while two participants also mentioned including family members and other professionals working with the child. One of the participants stated, “though the presence of peers and teachers are very important, it is often not feasible to work with them”.
vi. *Language used for pragmatic language assessment for preschoolers:* In terms of the language used for the assessment of pragmatic language, a majority (82%) of the SLPs reported assessing the same in the preschooler’s native language, 48% assessed in all languages the child was fluent in, while22% assessed in the language which was deemed as the medium of instruction at their school.
vii. *Source of normative data on pragmatic language of preschoolers:* For the normative data on the development of pragmatic language among Indian preschoolers, SLPs relied on scholarly research articles (65%), discussion with colleagues (61%), their own experience and judgment (59%), books on child language development (57%), and internet (blogs, webpages, social media) (33%). Most participants (86%) used more than one source to obtain the normative data on pragmatic language. Five percent of the SLPs reported using all the five sources to obtain information on pragmatic language development among Indian preschoolers.

**Table 4.** Different assessment tools used by the study participants.

Sl no.	Assessment tools	Age range	Participants ( *n*)
1.	Clinical Evaluation of Language Fundamentals - 4 ^th^, 5 ^th^ edition (Pragmatics profile section) ( Semel et al., 2004; Wiig et al., 2013)	5 – 21.11 years	7
2.	Speech and Language Development Chart – 2 ^nd^, 3 ^rd^ edition ( Gard et al., 1993; Kipping et al., 2012)	Birth – 7 years	4
3.	Pragmatics Profile of Everyday Communication Skills in Children (preschool, school-age version) ( Dewart & Summers, 1995)	Birth - 4 years, 5-10 years	4
4.	Social Communication Skills – The Pragmatics Checklist ( Goberis, 1999)	2 – 5 years	3
5.	Pragmatic Language Skills Inventory ( Gilliam & Miller, 2006)	5 – 12.11 years	2
6.	Test of Pragmatic Language – 2 ^nd^ edition ( Phelps-Terasaki & Phelps-Gunn, 2007)	6 – 18.11 years	2
7.	Communication DEALL Developmental Checklist - social skills section ( Karanth, 2007)	Birth – 6 years	2
8.	McGinnis Pragmatic Skills Checklist ( McGinnis, 1999)	Children	1
9.	Linguistic Profile Test ( Karanth, 1980)	6 – 10+ years	1
10.	Test of Narrative Language – 2 ^nd^ edition ( Gillam & Pearson, 2017)	5 – 15.11 years	1
11.	Children's Communication Checklist – 2 ^nd^ edition ( Bishop, 2003)	4 – 16.11 years	1
12.	Checklist for Assessment of Pragmatics in Preschoolers ( Deosthalee & Waknis, 2020)	Birth – 4 years	1
13.	Communication matrix ( Rowland, 2013)	Birth – 21 years	1
14.	Indian Scale for Assessment of Autism ( National Institute for the Mentally Handicapped, 2009)	3 – 22 years	1
15.	Receptive-Expressive Emergent Language Test	Birth – 3 years	1
16.	Childhood Autism Rating Scale ( Schopler et al., 1988)	> 2 years	1
17.	Verbal Behavior Milestones Assessment and Placement Program ( Sundberg, 2008)	Birth – 4 years	1
18.	Test of Pragmatic Skills ( Shulman, 1986)	3 – 8.11 years	1
19.	Preschool Language Scales – 5 ^th^ edition ( Zimmerman et al., 2011)	Birth – 7.11 years	1
20.	Other (unidentified tools: MECD, PLA, Test of early milestones, Pragmatic assessment)	-	4

### Barriers and facilitators towards pragmatic language assessment

**Figure 4.**
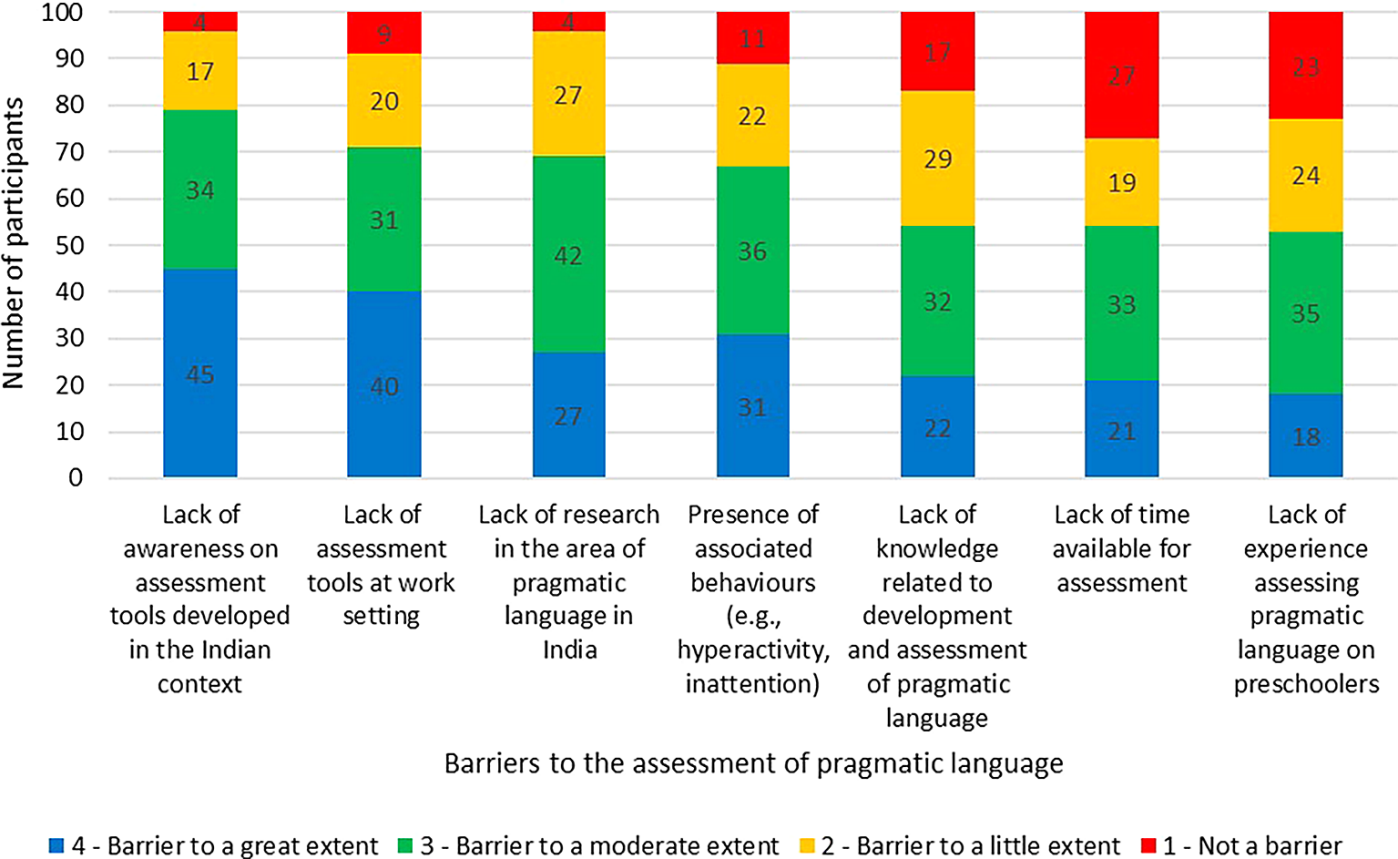
Barriers and its influence on the pragmatic language assessment in preschoolers.

The barriers and its influence on the assessment of pragmatic language in preschoolers was rated on a four-point Likert rating scale. The barriers and the extent of its influence (average mean) on the assessment routine were the lack of awareness on assessment tools developed in the Indian context (3.2), the lack of assessment tools at work setting (3.02), the lack of research in the area of pragmatic language in India (2.92), the presence of associated behaviours (e.g., hyperactivity, inattention) (2.87), the lack of knowledge related to development and assessment of pragmatic language (2.59), the lack of time available for assessment (2.48), and the lack of experience assessing pragmatic language on preschoolers (2.48). The barriers and its influence on the assessment of pragmatic language is graphically presented in Figure 4.Other barriers perceived to limit the participants from effectively conducting an assessment of pragmatic language among preschoolers were stated as, “incomplete profiling of the child’s pragmatic language due to non-feasibility of assessing in multiple settings such as child’s home, school, and situations such as peer group etc.”, “biased parental reporting and poor reliability of the information provided by parents”, “lack of access to resources specific to the pragmatic language development among Indian children”, “lack of importance given to pragmatics as a part of language in general, during the graduate teaching or even during later clinical practice”, and, “there are many other areas to be assessed so assessing each in detail would be difficult”.

**Figure 5.**
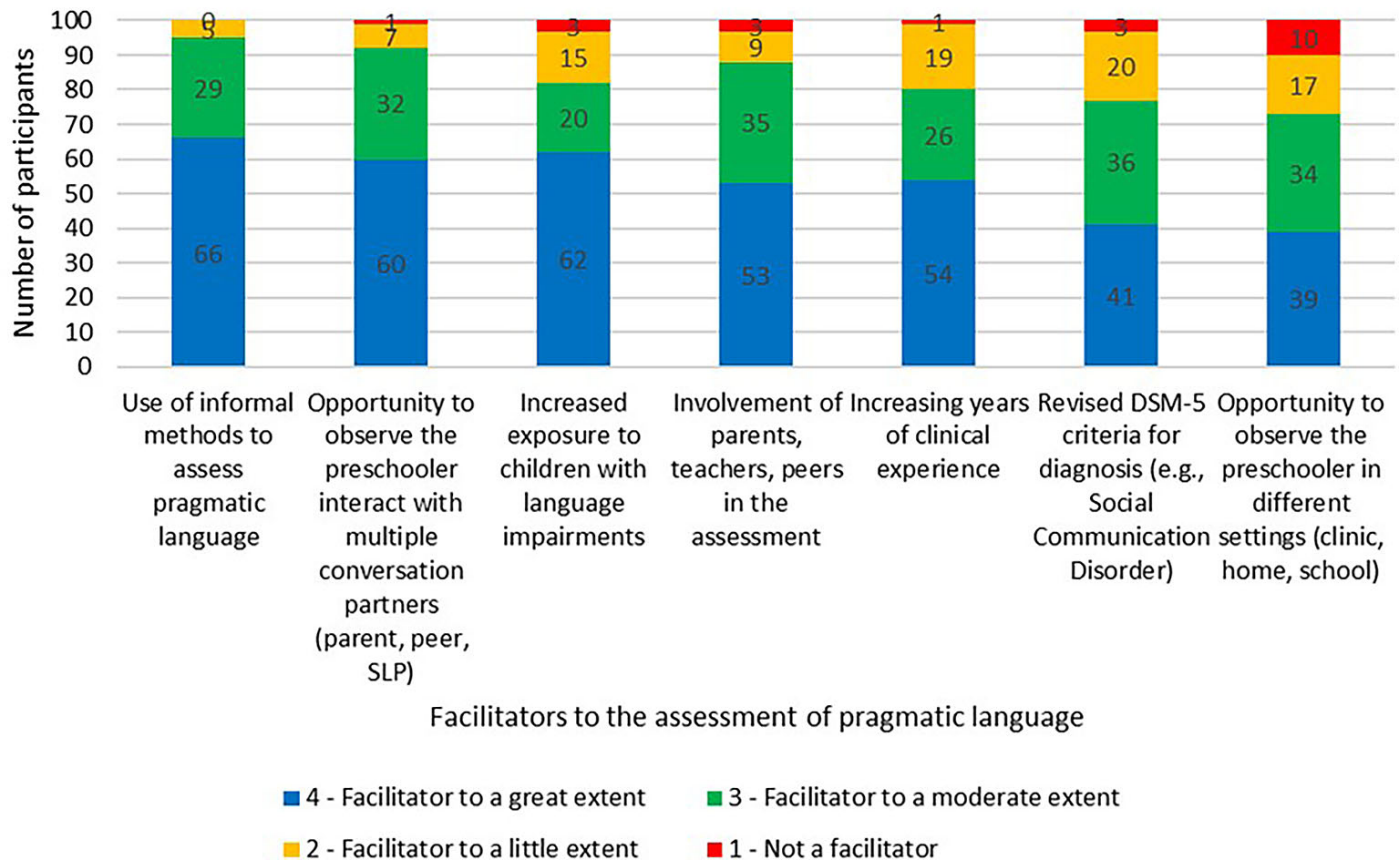
Facilitators and its influence on the pragmatic language assessment in preschoolers.

The list of facilitators and its influence on the assessment of pragmatic language was rated on a four-point Likert rating scale. The facilitators and the extent of its influence (average mean) on the assessment routine were the use of informal methods to assess pragmatic language (3.61), the opportunity to observe the preschooler interact with multiple conversation partners (parent, peer, SLP) (3.51), the increased exposure to children with language impairments (3.41), the involvement of parents, teachers, peers in the assessment (3.38), the increasing years of clinical experience (3.33), the revised DSM-5 criteria for diagnosis (e.g., Social Communication Disorder) (3.15), and the opportunity to observe the preschooler in different settings such as at clinic, home, school (3.02). The facilitators along with its influence on the assessment of pragmatic language is graphically presented in Figure 5. Other facilitators stated by the SLPs included having discussion with colleagues pertaining to the same and having more interaction time with the child.

### Self-appraisal of knowledge, skills and overall practices

SLPs’ knowledge, skill, and overall practice towards assessment of pragmatic language among preschoolers were identified on a five-point Likert rating scale of quality, where 1 = poor, 2 = below average, 3 = average, 4 = good, and 5 = excellent. Knowledge of SLPs about the development of pragmatic language among preschoolers obtained higher mean (3.37) when compared to knowledge of available assessment methods (3.01) which in fact was observed to have obtained the least mean score across any item on the self-appraisal section. The skills of SLPs to identify pragmatic impairment/deficits among preschoolers obtained higher mean (3.66) when compared to the skills to perform assessment of pragmatic language on preschoolers (3.46). In terms of self-appraising SLPs practices, the practice of SLPs to perform assessment of pragmatic language among preschoolers obtained a higher mean (3.48) when compared to the overall practice towards the assessment of pragmatic language among preschoolers(3.41). Overall, the results indicate knowledge domain to be significantly poorer compared to the skills and overall practice domains; and in specific, the knowledge, skill and practices related to assessment of pragmatic language was poorer. The meanand percentage of participants’ rating for each item of self-appraisal is represented in Table 5.

**Table 5.** Self-appraisal of knowledge, skill and overall practice of the study participants.

Domain	Item	Mean rating score	Participants rating (%)				
			5	4	3	2	1
Knowledge	Development of pragmatic language among preschoolers	3.37	5	38	47	9	1
	Assessment methods available	3.01	-	22	60	15	3
Skill	Perform assessment of pragmatic language on preschoolers	3.46	3	50	39	6	2
	Identify pragmatic impairment/deficits among preschoolers	3.66	10	53	31	5	1
Practice	Practice to perform assessment of pragmatic language among preschoolers	3.48	7	46	38	6	3
	Overall practice towards the assessment of pragmatic language among preschoolers	3.41	3	45	44	6	2

1 - Poor;

2 - Below average;

3 - Average;

4 - Good;

5 - Excellent;

*n* = frequency.

## Discussion

The study aimed to explore SLP practices towards the assessment of pragmatic language among Indian preschoolers, the barriers, facilitators, and self-appraisal of knowledge, skills, and overall practices. Using a survey method, the study gathered information from 100 SLPs working with preschool children across 13 states of India, providing a comprehensive understanding of the prevalent assessment practices. The main findings on the approaches, barriers, facilitators, and self-appraisals based on the assessment of pragmatic language of preschoolers are discussed below.

### Approaches to pragmatic language assessment

*Caseload representation and choice of pragmatic language assessment*

Preschoolers with ASD were encountered by a majority (95) of SLPs in the study. These findings are consistent with studies reported from India ( Mendonsa & Tiwari, 2018), United States of America ( American Speech-Language-Hearing Association, 2022; Izaryk et al., 2021), Canada ( Binns et al., 2022), and other countries globally ( Gillon et al., 2017). With the increase in global prevalence rates of ASD ( Zeidan et al., 2022), early age of identification ( Brett et al., 2016; Centers for Disease Control and Prevention, 2019), and intervention ( Gillon et al., 2017), it becomes foreseeable for preschoolers with ASD to be encountered more frequently by SLPs ( Binns et al., 2022) as evident in the findings of this study. While 73.68% (70/95) of SLPs assessed for pragmatic language on this population, 26.32% (25/95) failed to do so. Since ASD is primarily characterised by social communication and pragmatic language deficits ( Swineford et al., 2014), an assessment of pragmatic language becomes crucial. On similar lines, preschoolers with DLD being commonly reported in India ( Binu et al., 2014; Sidhu et al., 2013), have been encountered by the SLPs (with an equivalent majority as ASD). This indicates ASD and DLD to be the two most prevalent diagnoses in the caseloads of SLPs working with preschool age children in India. In terms of assessment, 69.47% (66/95) of SLPs evaluated pragmatic language among preschoolers with DLD, while 30.53% (29/95) failed to do so. Although not primarily a social or pragmatic language disorder, assessing pragmatic language becomes crucial due to strong evidence of pragmatic language deficits among preschoolers with DLD ( Vassiliu et al., 2023; Wong et al., 2022). Concurrently, preschoolers with a diagnosis of ADHD were encountered by 85 SLPs. Among them, 71.76% (61/85) assessed for pragmatic language, except for the 28.24% (24/85). ADHD being characterised with poor topic maintenance, incoherent narration and other pragmatic language deficits ( Carruthers et al., 2022; Norbury et al., 2014; Norbury & Bishop, 2003), it becomes important to assess the same. In comparison with other neurodevelopmental disorders (GDD, DS, CP, CLP and others) (on whom SLPs assessed pragmatic language in less than 50% of the total number encountered), the SLPs conducted pragmatic language assessment on a relatively larger percentage [59.38% (38/64)] of preschoolers with ID. Overall, it was found that SLPs prioritized the assessment of pragmatic language on preschoolers diagnosed with ASD, DLD and ADHD. This preference may stem from ASD’s recognition as a social communication disorder, the association of ADHD with pragmatic language impairments, and the relatively higher prevalence of DLD within SLPs caseloads. Over half of the participants (53%) reported conducting routine assessments of pragmatic language on every preschooler in their caseload, while 47% assessed only a proportion of them. Majority (71%) of the SLPs preferred only screening as opposed to conducting diagnostic or in-depth evaluation, possibly due to unavailability or the absence of specific diagnostic tools for Indian preschoolers.

*Assessment of different aspects of pragmatic language*

In the current study, most SLPs assessed multiple aspects of pragmatic language such as the way of using language for different purposes, conversation abilities, narrative abilities, the way of adapting language according to listener (considering partner’s age and relationship) and situation, while one-third of the participants assessed all these aspects. It is encouraging to note that all SLPs from the study reported of assessing multiple aspects of pragmatic language among preschoolers. This comprehensive approach is crucial since pragmatic language skills are foundational for effective communication and social interaction ( Dillon et al., 2021; Green et al., 2014; Toe et al., 2020). It was observed that the aspect related to the way of adapting language according to listener and situation were comparatively less assessed by SLPs. This could be attributed to the limited availability of communication partners of varying ages and roles, as well as the challenge of replicating situations in a testing environment respectively. Regardless of the clinical diagnosis, each aspect of a preschooler’s pragmatic language should be assessed ( Gillon et al., 2017). By ensuring multiple aspects of pragmatic language to be assessed, SLPs obtain a comprehensive profile of the child’s abilities, providing more effective and individualized support, ultimately enhancing the child's overall communication abilities ( Adams, 2002; Norbury & Bishop, 2003).

*Methods to assess pragmatic language*

Using appropriate approaches to assess pragmatic language among children is crucial ( Wong et al., 2022). While each method has its own strengths and weaknesses, the most ideal approach remains questionable. Professional guidelines suggest incorporating standardized tools as an integral part of the diagnostic process ( Betz et al., 2013). However, the dynamic nature of pragmatic language necessitates evaluation across different individuals, situations and environments reflective of natural everyday life ( Denman et al., 2023), a requirement that standardized tools are unable to meet. Reasonably, only 38% of the participants have reported of using standardized tools for the assessment of pragmatic language, concurrent with best practices that warn the cautious use of formal assessment tools for several reasons ( Brinton & Fujiki, 2019; Izaryk et al., 2021; Norbury et al., 2014; O’Neill, 2014; Timler, 2018). Firstly, due to its inability to capture the dynamic and context-dependent nature of social communication or pragmatics; and secondly, due to the lack of ecological validity and difficulty in terms of standardizing. Furthermore, anecdotal evidence based on clinical reports suggest a mismatch between children's pragmatic knowledge and their ability to apply the same in real-life situations thus recommending clinical evaluations to incorporate in-person assessments ( Carruthers et al., 2022).

Traditionally, natural observation methods were most preferred, providing an understanding of pragmatic language skills as used by children in real-life situations ( Wong et al., 2022). This preference has evidently continued as majority of the participants reported of using natural observations (80%), parent-child interactions (90%), and peer-child interactions (55%). Researchers in India commonly use these methods at child’s home, school or test environment to study the development of pragmatic language ( Harinath & Raghunathan, 2017; Shilpashri & Chengappa, 2015; Shilpashri & Shyamala, 2020). In clinical practice, majority (64%) of the SLPs reported assessing preschoolers’ pragmatic language in more than one environment, however, 36% assessed only in a single environment. Most of them assessed pragmatic language of preschoolers at the test/consultation room compared to other settings such as home, preschool, etc. Though assessing in multiple settings is recommended ( Binns et al., 2022; Tulviste & Schults, 2023), the constraints of increasing caseloads and lack of resources, makes it challenging for SLPs to visit different environments of preschoolers and conduct observations in-person. Alternatively, SLPs have begun requesting video recordings of preschoolers in these environments. In the present study, three SLPs reported requesting such recordings.

In the absence of the provision to visit in-person or obtain video recordings of preschoolers in different environments, information about the child can be gained from parents, caregivers, and teachers through interviews, checklists or questionnaires. Having a number of individuals of different age and relation during the assessment of pragmatic language provides a scope to assess for specific aspects of pragmatic language such as the way of adapting language to different listeners and also situations ( Manohar et al., 2024). However, these methods are prone to recall bias, positive praise, and manipulation of information ( Krupa et al., 2019). According to studies conducted in Canada, United States of America and United Kingdom, parents desire to be a part of the evaluation so as to communicate, collaborate, be informed, share knowledge, and be able to build a positive relationship with their child ( Gillon et al., 2017; Starr & Foy, 2012). In this study, majority of the SLPs used methods that require gathering information from parents and caregivers (81%) than teachers (25%), possibly due to the lack of questionnaires developed in India for teachers, difficulty in reaching out to them (as also reported by one of the participants in the study), convenience (parents or caregivers typically accompany their children for consultation) and economy of time ( Krupa et al., 2019). Although parents tend to have a broad, detailed, and naturalistic view of their child’s pragmatic language skills, having teachers’ systematic observations of the child in preschool environment through reports and/or interviews provide a holistic perspective towards pragmatic language assessment ( Tulviste & Schults, 2023). Additionally, involving siblings and peers can offer valuable insights into the nuances of pragmatic language within similar age group ( Tulviste & Schults, 2023). While this may be feasible in certain clinical settings, it may not be possible in others.

Use of informal methods for the assessment of pragmatic language are often reported as time consuming and challenging to apply in clinical contexts ( Toe et al., 2020). Despite the drawback, the data from this survey revealed majority (92%) of the SLPs to have used informal tasks and activities to assess pragmatic language among preschoolers ( Binns et al., 2022; Izaryk et al., 2021). Further to corroborate the findings, it was observed that time was not regarded as a major factor for the assessment of pragmatic language among preschoolers. On the contrary, the use of informal means of assessment was reported as a facilitator to a greater extent by majority of the participants (66%). Furthermore, from the open-ended responses to the survey, the scope of using informal tasks and activities seemed potent, with a participant stating “there are a lot of informal tasks and activities to even mention” and another mentioning, “there is no one particular activity; any activity depending on the child's interest can be used”.

Typically, informal tasks or activities are staged situations that mimic a naturalistic context, where the SLP and child involves in real-life social interactions. This enables the SLP to create multiple opportunities and observe the child’s pragmatic language competency in a naturally created social context rather than solely relying on information from others. Informal methods have incorporated stories ( Anil et al., 2021; Loukusa et al., 2018), conversations ( Wetherell et al., 2007), narrations (personal narratives, story narrations) and other tasks or activities into structured or unstructured interactions during the assessment of pragmatic language. Using such tasks allows researchers and SLPs to observe and assess language in an ecological way, as used by the child in everyday situations ( Wetherell et al., 2007). The potential of using informal tasks and activities to provide insight into the preschoolers pragmatic language in action highlights the future assessment opportunities for SLPs in the absence of culturally developed standardized tools, and constraints of assessing the child in multiple contexts ( Izaryk et al., 2021).

It is important that the materials used during the assessment of pragmatic language among preschoolers are age-appropriate, age-relevant, versatile and can provide scope to elicit different pragmatic language skills ( Manohar et al., 2024). It becomes equally important to use materials that gain child’s interest, attention, and participation ( Ferjencik, 2019). From the results of the study, toys were reported to be the most frequently used material. Beyond their common availability at a SLPs’ clinic for engagement and reinforcement purposes, toys are highly useful for creating numerous contexts to assess various pragmatic language skills thus making it most suitable during the assessment of pragmatic language among preschoolers ( Ferjencik, 2019). Other specific toys reported and found to be used in studies include pictures, small characters, plastic animals ( Loukusa et al., 2018), blocks, phones, puppets ( Shulman, 1986), utensils, cars, balls, bubbles, papers, crayons, books, boxes, eatables and others ( Ferjencik, 2019; Zimmerman et al., 2011). Additionally, two participants reported using videos during the assessment of pragmatics in the preschoolers. Although the specific purpose whether to capture and present the child's language use in situations beyond those directly observed by the SLP, or to display animated or recorded social situations to assess child's pragmatic language competency ( Lavi et al., 2016) remains unknown; nonetheless, the potential of using videos in the assessment of pragmatic language is increasing.

*Awareness and use of assessment tools for pragmatic language assessment*

Majority (61%) of the SLPs reported of being unaware of Indian developed tools for the assessment on pragmatic language in preschoolers. Recently there have been no Indian tools developed specifically to assess pragmatic language of preschool age children. The developed tools in the past included the Developmental Protocol for Pragmatics ( Dheepa & Shyamala, 2005), and Test of Pragmatics ( Sundaram & Karanth, 1994) [an adaptation of Test of Pragmatic Skills ( Shulman, 1986)]. Both these tools remain unpublished, confined to the institutional archives making it inaccessible to SLPs, and are now considered obsolete.

In terms of using tools for the pragmatic language assessment, majority of the participants reported western-standardized tools, with CELF being most commonly used by SLPs in the current study and also globally ( Betz et al., 2013; Gillon et al., 2017; Izaryk et al., 2021). Nonetheless, SLPs in India commonly used un-adapted versions of the western tools on preschoolers for the assessment of pragmatic language due to the unavailability and inaccessibility to indigenous tools. Though these tools have been standardized and proven efficient in identifying deficits in pragmatic language, they remain inapplicable in the Indian context due to the cultural and linguistic influence unless adapted otherwise. The only Indian developed tools reported to be used by the participants of the current study were the CDDC, LPT, Indian Scale for Assessment of Autism (ISAA), and CAPP. CDDC is a criterion referenced checklist (administered on children from birth to six years), that assesses developmental skills on eight domains of which one being social skills. LPT involves the assessment of phonology, syntax, and semantics with a small part of discourse ( Suchitra & Karanth, 1989). Although reported to be used by one of the participants in this study, this tool (LPT) does not involve the assessment of pragmatic language per se nor assess preschool age children ( Mendhakar & Priyadarshi, 2018). ISAA is an objective assessment tool (administered on children of 3 years and above until 22 years) that uses a 5-point rating scale to identify deficits on six domains of which one included social relationship and reciprocity. A recent study by Manohar et al. (2024), revealed ISAA to be unable to accurately capture the profile of children having good expressive language abilities but lacking social-communication skills due to pragmatic language impairment. Further, the tool has demonstrated suboptimal performance among children <5 years of age. CAPP is a modification to an earlier adaptation of the preschool version of PPECS to Marathi speaking children in India. This tool uses a parental or caregiver checklist to obtain information on four sections: interaction and conversation, communicative functions, contextual variation and response to communication on Marathi speaking children between birth and 4 years, excluding children between 5 and 6 years of age. It becomes important to consider the publication year of tools for assessment, as tools published over 15 years ago (except for CAPP) may not be suitable for use, as their stimulus items might no longer be socially relevant or commonly encountered ( Betz et al., 2013; Salvia et al., 2017).

The Indian developed tools reported by participants in the study were either western developed (CELF, SLDC, PLS) or adapted versions (CAPP), comprehensive language test with a minor portion on pragmatics or social communication (CDDC), unsuitable to the age of the child [Linguistic Profile Test (LPT)] or specifically developed for children with ASD focusing on social relationship and reciprocity, and not pragmatic language abilities (ISAA). It was alarming to observe SLPs report of western tools (PLS, TOPL) when specifically enquired about Indian developed tools indicating the lack of knowledge and awareness about pragmatic tools and its targeted audience.

With over 19 assessment tools listed by the current study participants, the findings reinforce the critical need for the development of indigenous tools to identify pragmatic language impairments among preschoolers, as also iterated by Binns et al. (2022). In developing countries such as India where SLPs manage with limited resources, locally adapted tools requires to be developed and accessible ( Chakraborty et al., 2015). Tools particularly targeting the assessment of pragmatics in India are less, compared to the other aspects of language ( Landa, 2005), as also evidenced by participants in the current study. Given the limited focus on the assessment of pragmatic skills in India, there is a dire need to develop culturally sensitive pragmatic assessment tools ( Anil et al., 2021). As stated “tools need not be extensive but should be comprehensive to provide sufficient opportunities for children to express their abilities” ( Krupa et al., 2019).

*Language used for the assessment of pragmatic language*

Since bilingualism and multilingualism are a common aspect in India, conducting direct assessments on one-to-one basis raises questions on the language used to assess the child. An evaluation in both the languages is highly recommended when considering children from a bilingual background ( Patla et al., 2023; Tulviste & Schults, 2023). With the first language being the child’s native language, the second language will often be a locally spoken language, or the language used as a medium of instruction at the preschool. The results of the study revealed majority of the SLPs to have used the preschooler’s native language for the assessment, while 32% assessed pragmatic language in both (native and medium of instruction at preschool). The effective implementation of this practice is limited to the wide variety of languages spoken across India, the odds of finding SLPs or translators proficient in those languages, and the unavailability of tools ( Valliappan et al., 2022). As a contingency measure, questionnaires and checklists should include items that assess the pragmatic aspect regardless of the language spoken by the child ( Pesco & O’Neill, 2023).

*Sources of normative data on pragmatic language*

The United States ranked the highest, followed by the United Kingdom and China, in producing research on pragmatic language development ( Alduais et al., 2022). As a result, it is possible that the clinical practices in India are largely an adaptation of Western approaches. Given India's cultural and linguistic diversity, it is essential to develop indigenous theoretical and practical frameworks for the development, assessment, and intervention of pragmatic language. However, despite the limited resources with respect to the development and assessment of pragmatic language among Indian preschoolers ( Patla et al., 2023), there is evidence that SLPs frequently engage in research-informed practices. Most participants referred to research articles, books and collaborated with colleagues. Although around 59% of the SLPs relied on their own experience and judgment (though being an important aspect in the clinical decision-making), 33% reported using information platforms such as the webpages, blogs and social media to educate on the same.

SLPs being professionals actively involved in the identification and provision of intervention among children with various neurodevelopmental disorders, it becomes essential for them to be updated with the developments in the field ( Mendonsa & Tiwari, 2018). Disseminating information on newly published research articles, assessment tools, methods, interventions or advances related to pragmatic language through creative means such as posts, posters, flyers, and other printables on social media, social networks (professional groups), and other platforms becomes the need of the hour.

### Barriers and facilitators towards pragmatic language assessment

The barriers largely influencing the assessment included the lack of awareness on assessment tools developed in the Indian context. Though it is encouraging to observe SLPs considering their unawareness as a barrier, there have not been any Indian tools recently developed pragmatic language assessment, with the exception of CAPP ( Deosthalee & Waknis, 2020). Other barriers similarly influencing the assessment included the lack of assessment tools at work setting, and lack of research in pragmatic language in India. Considering the reports from the SLPs of the current study, it has become evident that these barriers have influenced the assessment practices resulting in them using the available tools irrespective of the cultural or language background of the child ( Valliappan et al., 2022). This could potentially lead to over-estimating the extent of impairments due to the variation between the Western and Indian English.

Some of the facilitators influencing the assessment to a greater extent included, the use of informal methods to assess pragmatic language, opportunity to observe preschoolers interact with different conversation partners (parent, peer, SLP), increased exposure to children with language impairments, and the involvement of parents, teachers, peers in the assessment. Although the use of informal approaches are often considered time-consuming ( Toe et al., 2020), time was considered to only minimally influence the SLPs of the current study. However, it is important to consider prolonged assessments to fatigue the preschoolers influencing their performance ( Betz et al., 2013). Hence it becomes important that a set of informal tasks or activities are pre-set and employed in an engaging manner with the preschooler. Involving multiple communication partners and providing an opportunity to interact with each of them provides a holistic outlook of the preschooler’s pragmatic language. Having an increased exposure with preschoolers of different diagnoses leads to experiential learning for SLPs, thereby influencing their decision-making abilities.

### Self-appraisal of knowledge, skills and overall practices

SLPs self-appraised their knowledge on the methods for pragmatic language assessment as being poorer compared to the developmental patterns of pragmatic language among preschoolers. Similarly, the skills to assess pragmatic language was felt to be poorer compared to the skills to identify pragmatic impairments among preschoolers. The SLPs collectively obtained poorer scores on items related to the assessment of pragmatic language, attributing it to the absence of guidelines and best practice recommendations for pragmatic language assessment for Indian SLPs. Overall, the knowledge aspects obtained poor scores compared to skill and practice. SLPs can identify their strengths and weaknesses, and adjust their learning and practices accordingly through self-appraisal of own performances ( Yan, 2020). It becomes important for SLPs to become educated on the development of culturally and linguistically appropriate assessment tools, different methods, relevant and recent research work, during their education and profession. SLPs require to be constantly updated through newsletters, broadcasts, e-mails, scientific seminars, webinars, conferences, etc. Only when SLPs are aware of available tools would they procure and use the same for clinical practice.

### Strengths and weaknesses

The findings from the current study provide valuable insights on SLPs’ approaches to pragmatic language assessment of preschoolers in the absence of best practices or clinical guidelines in India. The prevalent SLP practices included the preference for screening over in-depth evaluations, assessment of multiple aspects of pragmatic language, using multiple methods (with majority using informal tasks and a small number using standardized tools including both western and Indian), inclusion of parents/caregivers to gain additional information about the child, use of preschooler’s native language to conduct assessment and mostly at the test room setting. SLPs will now be able to identify the extent to which their practices align or differ from the clinical practices of peer SLPs within the country. The result of the current study provides a greater understanding of the most prevalent and benefitting practices thereby directing SLPs towards effective pragmatic language assessment of preschoolers in India. Perspectives towards the barriers, facilitators, knowledge, skills and practices of SLPs are beneficial to clinicians, academicians and researchers in the field.

The study findings are limited to a small number of participants relative to the number of SLPs working with preschoolers in India. Although the survey link was posted on the ISHA website, a major number of SLPs participated from Karnataka, possibly due to the abundance of speech and hearing institutions in the state. In this regard, further surveys may consider using measures to obtain equal participation from SLPs across India. The present study attempted to minimize the possibility of recall bias by enquiring information based on the assessment practices over the past six months as opposed to longer intervals. However, as inherent to this type of data collection (survey), the information provided could not be verified.

### The way forward

Pragmatic language assessment among preschoolers using in-person observations require supplementing information from other evaluation methods (questionnaires, interviews or reports). Consistent with recommended practices for assessment ( Izaryk et al., 2021; Tager-Flusberg et al., 2009; Timler, 2018), the SLPs of the current study reported using multiple methods during the assessment process ( Binns et al., 2022). In the event of a discrepancy in the preschooler’s pragmatic language abilities observed with the information reported, the need for assessment data to be collected from multiple informants becomes crucial ( Sturrock et al., 2020). Gaining information from individuals (parents, caregivers, teachers, peers) with whom the preschooler spends significant time is utmost essential ( Farmer & Oliver, 2005; Tulviste & Schults, 2023; Wong et al., 2022). A sensible strategy would be to combine measures of pragmatic language from more than one source ( Charman, 2004). A multi-informant approach becomes valuable helping mitigate factors that may impact objectivity ( Manohar et al., 2024). The best practice to obtain a holistic pragmatic language profile of preschooler, should include the evaluation of the different aspects, using multiple methods inclusive of informal tasks and activities, involving members of the family and others significant communication partners to the child, and obtain information from multiple settings tailored to the preschooler ( Elleseff, 2015; Izaryk et al., 2021; Krupa et al., 2019). Using a range of assessment tools, inclusive of formal and informal measures derived from multiple sources and across different contexts, is important to enhance the validity of assessment results ( Binns et al., 2022; Tager-Flusberg et al., 2009). Ultimately, the results of this survey recommend SLPs to undertake a comprehensive approach to the pragmatic language assessment of preschoolers ( Gillon et al., 2017).

## Ethics

The study was approved by the Institutional Ethics Committee of Kasturba Medical College, Mangalore, Manipal Academy of Higher Education, India as a part of a doctoral study (IEC KMC MLR 12-2020/418) on 18-03-2021.

## Consent

Informed consent was obtained from the participants by voluntarily placing a tick (✓) on the checkbox prior to participation in the study since the survey was conducted online using Google Forms.

## Data Availability

Open Science Framework: Data sheet for ‘Approaches towards pragmatic language assessment in Indian pre-schoolers: A survey among speech-language pathologists’. https://doi.org/10.17605/OSF.IO/V4Q2Z ( Rasheeka et al., 2024) This project contains the following underlying data:
• Data sheet.xlsx (Participant demographics and responses to each section of the survey on separate sheets of a single Excel workbook) • Checklist for Reporting of Survey Studies (CROSS).docx (Completed checklist) Open Science Framework:Survey questions for ‘Approaches towards pragmatic language assessment in Indian pre-schoolers: A survey among speech-language pathologists’. https://doi.org/10.17605/OSF.IO/V4Q2Z ( Rasheeka et al., 2024) This project contains the following extended data:
• Survey questionnaire.docx (Survey questions in a word document) Data areavailable for access on Open Science Framework under the terms of the Creative Commons Zero “No rights reserved” data waiver (CC0 Public Domain Dedication).
