# Supplementary material for: Approaches towards pragmatic language assessment in Indian pre-schoolers: A survey among speech-language pathologists
Source: F1000Res. 2024 Aug 1;13:865. [Version 1] doi: 10.12688/f1000research.154514.1 (PMC11464962; doi:10.12688/f1000research.154514.1)
Supplement: Peer review history

# Reviewer report: Reviewer response for version 1

**DOI:** 10.5256/f1000research.169554.r322030

**Published:** 2024-10-09

**Authors:** Leslie Kokotek

**Affiliations:** 1 University of Cincinnati, Cincinnati, Ohio, USA

The introduction is well written, clearly cited, and provides good rationale underscoring the need for this study. There are a few minor typos in the introduction that should be corrected prior to going to print. 

 In the results section I think DLD more commonly refers to Developmental Language Disorder. This is the term used by ASHA so it may not be applicable to this context. There are also some spacing issues when reporting percentages and other numbers.  

 The figures are nicely done and help to efficiently visualize the qualitative data. 

 Ideally, I would like to see a slightly extended description on the content analysis and how the data were examined. 

 This statement right here " This indicates ASD and DLD to be the two most prevalent diagnoses in the caseloads of SLPs working with preschool age children in India" I think is too forward of a claim to make given that only 100 SLPs were surveyed. This claim needs to be scaled back. 

 The discussion is a bit dense and I think perhaps some of the information such as the information about recall bias for parents would be better suited in the introduction rather than the discussion section. However, this may be a personal preference and I think reasonable the manuscript could move forward with indexing either way.

Is the work clearly and accurately presented and does it cite the current literature?

Yes

If applicable, is the statistical analysis and its interpretation appropriate?

Yes

Are all the source data underlying the results available to ensure full reproducibility?

No source data required

Is the study design appropriate and is the work technically sound?

Yes

Are the conclusions drawn adequately supported by the results?

Partly

Are sufficient details of methods and analysis provided to allow replication by others?

Partly

Reviewer Expertise:

My areas of expertise include (but are not limited to): child speech-language development, communicative participation, functional communication, assessment psychometrics, multi-lingual/cultural research.

I confirm that I have read this submission and believe that I have an appropriate level of expertise to confirm that it is of an acceptable scientific standard, however I have significant reservations, as outlined above.

# Reviewer report: Reviewer response for version 1

**DOI:** 10.5256/f1000research.169554.r322026

**Published:** 2024-09-26

**Authors:** Pallavi Kelkar

**Affiliations:** 1 Bharati Vidyapeeth (Deemed to be University), Pune, Maharashtra, India

I thank the researchers and the journal for the opportunity to review this work. Overall, I think this paper is a necessary addition to literature in order to get a better perspective on the way assessment of pragmatics is conducted by speech-language pathologists in India. Barring a few additions that the method and discussion sections might require, I think that the paper is well-written. The objectives, results and conclusions are well-aligned with each other. The structure of the survey instrument reveals that the researchers exhaustively covered different aspects of assessment of pragmatics including barriers, facilitators and self-appraisal of the participants’ knowledge and skills. I have put down my comments and suggestions for specific sections of the paper below.

 I recommend using the phrase "assessment of pragmatics" in the title as well as throughout the paper. Although the researchers establish in the introduction itself that, ‘pragmatic language’ and ‘pragmatics’ both mean the same thing, use of the term "pragmatic language" might be misinterpreted as restricted to ‘language assessment’.  "Pragmatics" could better reflect the inclusion of communication, non-verbals, etc. especially for a reader who might not be very familiar with these terminologies, considering the wide readership of this journal.

 The abstract aptly conveys the essence of the study. The Introduction covers an extensive review of existing literature and makes a case for the present study. However, some of the phrases in the introduction might benefit from rewording after a general spelling and grammatical check. For example, “the listener’s” and not “the listener needs” on line 5 of the Introduction; a space missing between “is inclusive” in paragraph 3, line 5; “Globally there exist best practices…” and not “exists”, and similar minor edits. Similarly, some phrases, albeit conveying the right content, could be clearer to readers if reworded. For instance, page 4 line 11, “…required throughout learning and constant self-evaluation in an SLP..”. Rewording this as ".... required first during training to be an SLP and later, for evaluation of one's own performance as a professional" could make it clearer to the reader. A similar example is the last line of the Introduction that reads, “Therefore, the current study also aims to identify the barriers and facilitators towards the assessment of pragmatic language among pre-schoolers and identify the level of knowledge, skill and overall practice of SLPs towards their own assessment routines.” This does not connect well with the previous sentence, which talks about self-evaluation of knowledge and skills by SLPs. Along these lines, the Introduction as well as the rest of the document could benefit from an overall spelling and grammar check once over. Other than these minor suggestions, the Introduction does justice to the topic chosen for study, in my opinion.

 I had a few queries with reference to the Method section. Firstly, the sample largely comprised of SLPs from Karnataka. Although the researchers did acknowledge that as a limitation of the study, I would be interested to know if there were any particular challenges they faced in getting a sample more representative of different states in India. This would have added considerably to the study’s applicability across India.

 In the first line of the sub-section titled Survey questionnaire, the researchers state, “The questionnaire was developed… to survey SLP’s current approaches towards …assessment of pragmatic language…”. This description of the questionnaire does not do justice to the multiple areas it covers. I recommend rewording this description to better reflect the nature of the questionnaire and align with the aims of the study.

 The first line of the second paragraph under the same subheading mentions that the judges were five SLPs. It would be useful to know how many years of experience they had, and whether any of them had experience specific to construction and conduction of surveys. The last sentence of the same paragraph mentions, “…the final questionnaire was ready to be piloted among six SLPs to determine the item clarity, logical sequence of questions, and the response options across the questionnaire.” It would be great if there was a short paragraph that followed, sharing the results of this pilot.

 Line 3 of the subheading Data analysis mentions that for open ended questions, the responses were grouped into main categories. Was this grouping done independently by two or more researchers? During classification into categories was there 100 percent agreement between researchers? If not, how was this resolved? Mentioning these details could benefit replication of the survey in future.

 Lastly, in the questionnaire, under the section on self-appraisal by SLPs, I was not clear about the difference between “Practice to perform assessment of pragmatic language among pre-schoolers” and “Overall practice towards the assessment of pragmatic language among pre-schoolers” within the domain, Practice. Clarification of this difference could help readers interpret the results better.

 The results have been appropriately presented and the tables and graph convey all the necessary information. I would like to appreciate the researchers for their excellent choice of graphs for all of the results presented in the document. Using the right kind of graphs makes the findings very clear, even to a reader who might not have in-depth knowledge of the topic.

 In the discussion, the researchers make a good attempt at explaining the results and corroborating them with literature. However, an additional facet to the discussion could be to corroborate findings of different sections of the questionnaire within the study itself. In the last line under the section Approaches to pragmatic language assessment, they mention that most SLPs preferred screening, “probably due to unavailability of diagnostic assessment tools”.  Since they have data to support this point (dearth of published assessment instruments from India as apparent from the literature review and reference list, lack of assessment tools reported by respondents in the section on Barriers), that can be mentioned here as well. Other barriers such as lack of time, etc. that surfaced in the survey could also be discussed as possible reasons for preferring screening over assessment.

 On page 14, under Awareness and use of assessment tools for pragmatic language assessment, in the last sentence of the third paragraph, the researchers express alarm over the SLPs having mentioned western tools when the question specifically enquired the use of Indian tools. Despite this, the SLPs’ self-appraisal scores for knowledge, skills and practices were 3 or more. Were the SLPs accurate in their self- appraisal? Also, what could have been the reasons for their incorrectly reporting names of tests (western as Indian)? Did their self-appraisal of their skills and practice reflect in their responses to other sections of the questionnaire? Corroborating findings across different sections of the questionnaire in this manner could make the discussion richer.

 Another addition could be to discuss the responses obtained in the “Other” category under the subsection on assessment of different aspects of pragmatics. Some of the responses might reflect SLPs’ lack of perspective with regards to assessment of pragmatics. For instance, would “awareness of body proximity” be applicable to assessment of pre-schoolers? Similarly, would assessment of “a child’s personality” reflect a child’s understanding of pragmatics? A child might be an introvert but still communicate appropriately as the situation demands. These responses could be discussed in the discussion section, highlighting that lack of protocols and best practices might lead clinicians to assess aspects which might not come under the scope of pragmatic development of pre-schoolers.

 Minor additions and rephrasing in a few places could also benefit the discussion. Under the section on Barriers and facilitators to pragmatic language assessment, in the last sentence of the first paragraph the researchers mention that using Western instead of Indian tools could potentially lead to overestimation of the extent of impairment. I am not sure if it would always be a case of overestimation, because pragmatics is being assessed, and not syntax.“Inaccurate estimation” would be a better choice of phrase. Lastly, under the section titled “The way forward”, it would be great to have the researchers’ suggestions on what can be done to improve knowledge, skills and availability of resources for SLPs.

 I commend the authors on having taken a step towards streamlining the assessment process for diagnosis of pragmatic impairments among pre-schoolers. I hope this research inspires action taken for setting up assessment protocols and best practices for assessment of pragmatics by SLPs in India.

 Regards

 Pallavi Kelkar

Is the work clearly and accurately presented and does it cite the current literature?

Yes

If applicable, is the statistical analysis and its interpretation appropriate?

Not applicable

Are all the source data underlying the results available to ensure full reproducibility?

Yes

Is the study design appropriate and is the work technically sound?

Yes

Are the conclusions drawn adequately supported by the results?

Yes

Are sufficient details of methods and analysis provided to allow replication by others?

Partly

Reviewer Expertise:

Speech and language development, early detection of language delays, fluency disorders, professional voice

I confirm that I have read this submission and believe that I have an appropriate level of expertise to confirm that it is of an acceptable scientific standard, however I have significant reservations, as outlined above.

# Reviewer report: Reviewer response for version 1

**DOI:** 10.5256/f1000research.169554.r310418

**Published:** 2024-08-07

**Authors:** Ahmed Alduais

**Affiliations:** 1 University of Verona, Verona, Italy

Dear Colleagues, 

 Thanks for sharing your paper about pragmatic language assessment in Indian preschoolers. I have enjoyed reading your paper which seems to be a well-conducted piece of research. 

 The introduction covers all the basic and needed topics related to the examined areas with recent and classic studies in the field. However, I suggest the authors conclude their introduction with stating the study aims or questions. 

 The methods seem to be average but could be upgraded by applying some minor changes. I suggest using the typical headings including sample, design, measures (validity and reliability of the used measures) and procedure (data collection, data analysis and ethical considerations). 

 The results section is excellent, so I have no comment on this section. 

 The same applied to the discussion and the rest of the manuscript. 

 I do wish you all the best in applying these minor revisions.

Is the work clearly and accurately presented and does it cite the current literature?

Yes

If applicable, is the statistical analysis and its interpretation appropriate?

Yes

Are all the source data underlying the results available to ensure full reproducibility?

Yes

Is the study design appropriate and is the work technically sound?

Yes

Are the conclusions drawn adequately supported by the results?

Yes

Are sufficient details of methods and analysis provided to allow replication by others?

Partly

Reviewer Expertise:

Clinical Linguistics, Developmental Psychology, Mixed Methods Research

I confirm that I have read this submission and believe that I have an appropriate level of expertise to confirm that it is of an acceptable scientific standard.
